# Induced Pluripotent Stem Cell-Derived Mesenchymal Stem Cells Hold Lower Heterogeneity and Great Promise in Biological Research and Clinical Applications

**DOI:** 10.3389/fcell.2021.716907

**Published:** 2021-09-30

**Authors:** Juan Zhang, Mingzhuang Chen, Jinqi Liao, Chongfei Chang, Yuqing Liu, Arshad Ahmed Padhiar, Yan Zhou, Guangqian Zhou

**Affiliations:** ^1^Guangdong Key Laboratory of Genomic Stability and Disease Prevention, Shenzhen Key Laboratory of Anti-Aging and Regenerative Medicine, Shenzhen Engineering Laboratory of Regenerative Technologies for Orthopedic Diseases, Department of Medical Cell Biology and Genetics, Health Science Center, Shenzhen University, Shenzhen, China; ^2^Guangdong Key Laboratory for Biomedical Measurements and Ultrasound Imaging, School of Biomedical Engineering, Shenzhen University Health Science Center, Shenzhen, China; ^3^Shenzhen University General Hospital, Shenzhen, China; ^4^Senotherapeutics Ltd., Hangzhou, China; ^5^Cheerland Danlun Biopharma Co., Ltd., Shenzhen, China; ^6^Lungene Biotech Ltd., Shenzhen, China; ^7^Central Laboratory, Longgang District People’s Hospital of Shenzhen and The Third Affiliated Hospital (Provisional) of The Chinese University of Hong Kong, Shenzhen, China

**Keywords:** mesenchymal stem cells (MSCs), induced pluripotent stem cells (iPSCs), iPSC-derived MSCs (iMSCs), heterogeneity, functional subpopulations, single cell transcriptomics, CRISPR/Cas9 screening

## Abstract

Mesenchymal stem cells (MSC) isolated from different tissue sources exhibit multiple biological effects and have shown promising therapeutic effects in a broad range of diseases. In order to fulfill their clinical applications in context of precision medicine, however, more detailed molecular characterization of diverse subgroups and standardized scalable production of certain functional subgroups would be highly desired. Thus far, the generation of induced pluripotent stem cell (iPSC)-derived MSC (iMSC) seems to provide the unique opportunity to solve most obstacles that currently exist to prevent the broad application of MSC as an advanced medicinal product. The features of iMSC include their single cell clone origins, and defined and controllable cultural conditions for their derivation and proliferation. Still, comprehensive research of the molecular and functional heterogeneity of iMSC, just like MSC from any other tissue types, would be required. Furthered on previous efforts on iMSC differentiation and expansion platform and transcriptomic studies, advantages of single cell multi-omics analysis and other up-to-dated technologies would be taken in order to elucidate the molecular origin and regulation of heterogeneity and to obtain iMSC subgroups homogeneous enough for particular clinical conditions. In this perspective, the current obstacles in MSC applications, the advantages of iMSC over MSC and their implications for biological research and clinical applications will be discussed.

## Graphical Abstract

Induced pluripotent stem cell-derived mesenchymal stem cells (iMSC) and primary MSC comparison: to show the advantages and applications of iMSC.

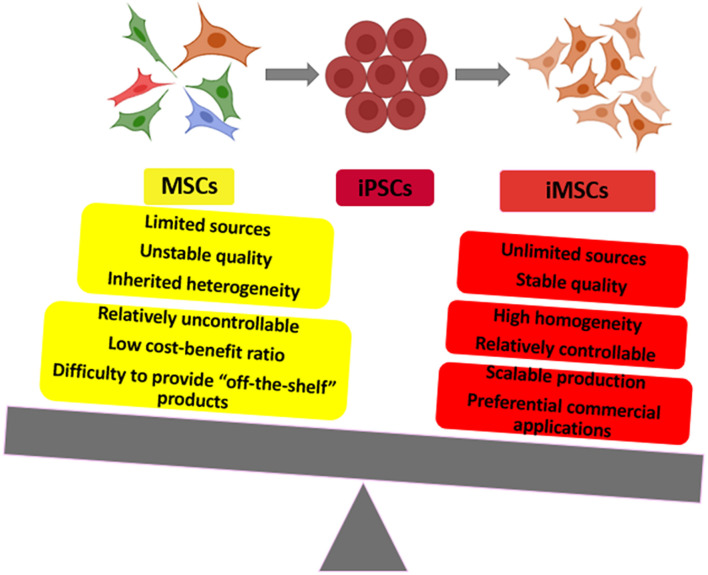

## Introduction

Mesenchymal stem cells (MSC) derived from organ tissues naturally possess high heterogeneity, which not only provides the basis for their multifunctional and complex activities, but also brings a hurdle to the reproducibility of MSC experiments and causes more variations and difficulties in the standardization and normalization to evaluate the safety and efficacy of MSC therapies (Yang et al., 2017; Chen et al., 2019; Zakrzewski et al., 2019; Wang et al., 2020).

Human induced pluripotent stem cells (iPSC) based on cell reprogramming technology have significantly improved the understanding of pluripotency maintenance and also provided infinite donor-related sources of specific individualized stem cells (Crow, 2019; McGrath et al., 2019; Chen et al., 2020). Importantly, iPSC-derived MSC (iMSC) still have these advantages. Compared with tissue-derived MSC, iMSC closely resemble their primary counterparts in morphology, immunophenotype, and three-lineage differentiation capacity while showing stronger regeneration ability in animal disease models (Ozay et al., 2019; Chang et al., 2020; Fernandez-Rebollo et al., 2020). Moreover, iPSC can be passed down indefinitely so that the sources of iMSC can be endless and iMSC induced from a single iPSC cell or clone are theoretically more homogeneous (Saetersmoen et al., 2019; Bloor et al., 2020).

Based on these characteristics, iPSC can provide stable and reliable sources of iMSC suitable for personalized treatment of the patients, and can also be utilized in infinitely scalable preparation and production. The qualities of the final products are sustainable, relatively controllable and can achieve the best therapeutic efficacy aimed at specific clinical diseases. In this perspective, we summarize the current research and discuss the potential contribution of iMSC in order to advance the field of iMSC-based biological research and clinical applications.

## Mesenchymal Stem Cell Heterogeneity in Their Biological Properties

Primary MSC could be isolated from several tissues such as bone marrow, adipose tissue perivascular fractions, Wharton’s jelly of human umbilical cord, umbilical cord blood and placenta, and regarding the cell lineage development, mesoderm and neural crest cells are the two main origin sources (Fukuta et al., 2014; Isern et al., 2014; Zhao and Ikeya, 2018). They play critical roles in regulating tissue functionalities and regeneration, possess multi-lineage differentiation potential and have been most widely used to treat tissue damage (Wong et al., 2013), organ degeneration (Orozco et al., 2013), aging (Ohta et al., 2020), immune-/inflammation-mediated diseases (Ciccocioppo et al., 2011; Connick et al., 2011; Tan et al., 2012; Gupta et al., 2019) in clinical applications whereas their diverse originations result in MSC heterogeneity and their various differential efficacy in biological activities.

Mesenchymal Stem Cell heterogeneity exists in multiple levels (e.g., age, metabolic status, hormone, genetic background, and pathophysiological condition), which lead to differences in nearly all the cell types of the body including MSC (Han et al., 2017; McLeod and Mauck, 2017; Wang et al., 2020). Secondly, primary MSC harvested from different tissues are naturally heterogeneous due to inherit tissue “memory” or be in different differentiation stages (McLeod and Mauck, 2017; An et al., 2018). Besides, it is worth mentioning that the micro-environment including anatomical structures and micromechanical factors and cellular “positional memory” varies and accounts for MSC heterogeneity (Sacco et al., 2019). In addition, various tissue separation methods and culture conditions (e.g., normal or hypoxia condition, low/high growing density) may also generate heterogeneous MSC subtypes or lead to their biased expansion toward specific types (Liu et al., 2015; Dhawan et al., 2016; Legzdina et al., 2016). Moreover, studies have shown that even from the same batch of MSC, their different colonies appear differences in proliferation ability and differentiation potential, which can be regarded as intra-clonal heterogeneity (Rennerfeldt and Van Vliet, 2016).

Earlier work found that both fat and bone marrow derived MSC contained two types of cells of different sizes which exhibited distinguished therapeutic effects in treating macular degeneration (Li et al., 2016; Wang et al., 2017). For a long time, people have used the MSC surface markers to distinguish their regenerative capacity. For example, the CD106^+^ MSC subgroup is likely to have superior angiogenesis and immune regulation functionalities (Yang et al., 2013). Now the MSC marker lists are growing and some of them, such as CD146, CD271, CD371, Notch1 and Stro1, have been used to screen cells capable for better migration, homing ability and preferential differentiation toward specific directions (such as endothelial cells, osteoblasts or chondrocytes, etc.) (James et al., 2015; Harkness et al., 2016; Najar et al., 2018). Accordingly, it seems that MSC subgroups with specific preferable capacity can be isolated and enriched by their distinguishable surface markers for better biological activities. However, the expression of these surface markers is not stable and there are conflicted reports in different laboratories. For example, studies have shown that MSC with high CD105 are more likely to mediate cartilage differentiation and regeneration whereas other laboratories cannot obtain consistent results (Su et al., 2015; Cleary et al., 2016).

Mesenchymal cells within different organs and tissues are comprised of functionally and devlopmentally diverse cell populations and they have been for long difficult to discriminate largely due to the lack of development and progenitor hierarchy-related surface markers. In-depth analysis of the derivation processes of iMSC in well-defined culture conditions has demonstrated the capacity of iPSC to generate different types of mesenchymal cell populations, including MSC, pericytes (PC) and other related cell types (Vodyanik et al., 2010; Slukvin and Kumar, 2018). In particular, successful identification of a common mesodermal progenitor for mesenchymal and endothelial cells, mesenchymoangioblast (MB), was an important milestone for well understanding mesenchymal progenitor cell development. MB is a transient status during the derivation of iMSC cultured in chemically defined conditions, existing on day 2 of differentiation and featured with APLNR, PDGFR and KDR expression, and dramatically reduced on day 3 and entirely disappear on day 4 of differentiation. MB is thereby suggested to be the earliest clonogenic mesodermal progenitors to mark the onset of endothelio- and mesenchymogenesis in iPSC cultures (Vodyanik et al., 2010; Slukvin and Kumar, 2018).

## Induced Pluripotent Stem Cell-Derived Mesenchymal Stem Cell Differentiation Systems Supply Extended Platforms for In-Depth Biological Research

To uncover the pathological mechanisms of genetic disorder, it is necessary to recapitulate the overall developmental process of specific physiological response or to acquire specific cell types with genetic mutations. However, due to limited access of patient samples of rare diseases and the difficulties to obtain and maintain primary cells, traditional sampling studies have confronted a lot of challenges. The emergence of iPSC redifferentiation platform may help to address these issues, and the generation of iMSC from patient-derived iPSC have enabled the functionality study of patient MSC which helps to address their roles in pathogenesis.

How to quickly produce a large number of iMSC with the same genetic background from iPSC is the first step of iMSC-mediated biological research. Several simple methods to convert iPSC to iMSC have been reported. For example, (1) Under the action of 10 ng/ml FGF2, 10 ng/ml recombinant human blood plate-derived growth factor (PDGF)-AB and 10 ng/ml EGF, combined with the flow cytometric sorting of CD24^–^CD105^+^, iMSC can be efficiently differentiated from iPSC (Lian et al., 2010). (2) Under the function of p38MAPK inhibitor SB203580, iPSC can form embryoid bodies in serum-free medium and iMSC with less tumorigenicity are gradually produced under the mediation of embryoid bodies (Wei et al., 2012). (3) Under the impact of TGF-β signaling pathway inhibitor SB431542, iPSC can be quickly differentiated into high-quality iMSC without involving the formation of embryoid bodies or requiring flow cytometric sorting or the participation of trophoblast cells (Chen et al., 2012; Zhao et al., 2015). In light of the technical limitations of the methods mentioned above related to iMSC heterogeneity, it would be highly speculable that scalable and cost-effective conversion of iPSC into functional, clinically usable iMSC in a unified training system still needs further research.

Fibrodysplasia ossificans progressive (FOP), with a point mutation a 617 G > A (R206H) in gene ACVR1, is a rare genetic disorder presenting abnormal constitutive activation of BMP signaling which further results in progressive ossification in soft tissues. The Matsumoto group first demonstrated that patient-iPSC derived MSC displayed increased mineral deposition and chondrogenesis, and further they developed a high-throughput screening system to study patient-iMSC differentiation and identified the causal roles of mTOR signaling for the aberrant chondrogenesis. Moreover, by targeting this found signaling pathway with rapamycin, normal chondrogenesis of patient-iMSC could be restored (Matsumoto et al., 2013; Hino et al., 2017).

Hutchinson Gilford Progeria syndrome (HGPS) is a rare but lethal genetic disorder. More than 90% of HGPS patients are caused by the production and accumulation of progerin induced by the silent G608G mutation in the LMNA gene (Eriksson et al., 2003; Harhouri et al., 2018; Piekarowicz et al., 2019). With the application of iMSC differentiation from Patient-derived iPSC, Alan’s group have demonstrated that G608 mutation causes proliferation defects of MSC, with robust progerin level, increased DNA damage and nuclear abnormality (Zhang et al., 2011). The other group further demonstrated that these patient-derived iMSC also showed premature osteogenic differentiation, and with the same system, they found seven compounds that could counteract with this premature differentiation bias and normalize their three lineage differentiation potentials (Lo Cicero et al., 2016).

Our team recently found that three HGPS patients in Chinese families carry a novel homozygous mutation c.1579C > T (R527C) in the LMNA gene, while the phenotypes of parents carrying the heterozygous mutation are normal. In order to investigate the causal effects of this novel mutation, we have generated iPSC (Chinese HGPS-iPSC) from these three patients by somatic cell reprogramming, corrected the mutation with CRISPR-mediated gene editing, and differentiated their corresponding iMSC. This allows us to explore the pathogenic mechanisms and to perform drug screening for this new type HGPS, while at the same time encouraging new ideas of aging and anti-aging research. Until now, we have demonstrated that this newly found mutation could also result in early cell cycle arrest in patient-derived iMSC compared with normal iMSC. We are now also expanding our understanding of the underlying mechanisms of hematopoiesis defect in patients with their iMSC.

Thus far, the therapeutic application of iPSC itself has been very limited whereas they are serving more as an ideal starting material for other cell types with higher direct therapeutic value. Also, CRISPR/Cas9-based gene editing on iMSC as a good experimental model system could be utilized to define the function and the mechanisms involved in stem cell differentiation and regeneration. Even further, if a sensible genetic variation occurs, the mutation could be corrected in single cells at the iPSC and/or iMSC level to meet the safety and efficacy standards.

In summary, the better controllable and monitorable process for generating iMSC has indeed provided certain unique opportunities for enhancing our understanding about MSC biology as well as widening the feasibility and application of MSC technologies.

## Multiple Induced Pluripotent Stem Cell-Derived Mesenchymal Stem Cell Advantages Over Primary Tissue-Originated Mesenchymal Stem Cell in Clinical Applications

Until now, there are more than 10,000 clinical trials of autologous and allogenic MSC intervention in various pathological conditions have been performed, with 40% of them have been completed in at least phase I study and 4% have been completed in phase III study^1^. For most of these clinical trials, the MSC products were isolated and generated from primary tissues, including bone marrow, adipose tissue vascular stromal fractions, Wharton’s jelly of human umbilical cord, umbilical cord blood and placenta.

In comparison, the first clinical trial with iPSC-derived products was performed in 2008. And now, the total number of iPSC-related clinical trials has risen to 54. Among these 54 trials, around 10 studies are now administering iPSC-derived cell therapeutics to human patients, and noteworthily, there is only one or two clinical trials with iPSC-derived MSC (see text footnote 1).

Although many attempts have been performed to demonstrate the therapeutic effects of primary MSC and there are currently more than ten types available commercial products for specific diseases (Paramsothy et al., 2017; Goto and Murata, 2018; Pellegrini et al., 2018), the United States Food and Drug Administration (FDA) has not approved any stem cell drugs (excluding orphan drugs and hematopoietic stem cell products) (see text footnote 1). This clearly reveals the difficulties to develop cell treatment drugs from primary MSC due to physiological limits.

The first challenge is to get massive amount of MSC from progenitor cell reservoirs. For most of the clinical trials, the suggested dose is 1–2 million cells per KG of body weight for every injection, which totally may require 100 million cells. As mentioned in their protocols, 2 ml of bone marrow could yield two million MSC in ideal condition, and to collect 100 million MSC, hence, it would take much more bone marrow fluid with expanded cell passages (Wakitani et al., 2011; Wang et al., 2013). Meanwhile, during the collection of autologous MSC, a lot of other issues have not been seriously evaluated. Firstly, the collection of bone marrow would cause interventional damage to the patients, and the culture of primary MSC would diminish their self-renewal capacity and cause cellular aging. Most importantly, the overall collection and expansion process may take several weeks, which is very time consuming and requires more labor work. Collectively, this is the most limiting factor for their further commercial applications, with much less possibility and much more difficulties to provide “off-the-shelf” products in reasonable cost, and for the allogenic cell infusion, it is pretty much the same case.

The second biggest issue would be the quality control of these primary MSC products between different batches isolated from different donors, different tissues or by different preparation methods, which, to some extent, is also due to lack of molecular standards for their characterization and purification. For autologous cell treatment, the MSC derived from patients may have diminished self-renewal capacity and therapeutic effects with age, hence, such treatment could only be applicable for specific range of people. And for the allogenic MSC derived from Wharton’s jelly of human umbilical cord, umbilical cord blood and placenta, it is also difficult to guarantee their efficacy beyond their inherited heterogeneity, although which may not affect their therapeutic effects. To make a conclusion, this uncertainty adds in the difficulties to evaluate their clinical efficacy and to popularize their applications.

Theoretically and practically, iMSC originated from one single iPSC clone are much more homogeneous than primary MSC isolated from diverse human tissues, and their biological performance are more stable and predictable since their molecular signature are barely changed among different batches. This idea has been carried out by the Cynata Therapeutics. In 2016, Cynata Therapeutics received approval to launch the world’s first formal trial of an allogeneic iPSC-derived cell product (CYP-001) which met all of its clinical endpoints and produced positive safety and efficacy data for the treatment of steroid-resistant acute graft-versus-host disease (GVHD). Consequently, Cynata is now advancing its iMSC into Phase II trials for the treatment of Corona Virus Disease (COVID)-19, GVHD and critical limb ischemia (CLI). It is also undertaking a massive Phase III trial that will utilize Cynata’s iMSC therapeutic, CYP-004, in 440 patients with osteoarthritis (OA) (Ozay et al., 2019; Saetersmoen et al., 2019; Bloor et al., 2020). Until now, the Cymerus TM technology platform and the C-Stem technology platform, established by Australian Cynata Therapeutics and French Treefrog Therapeutics, respectively, have managed to generate massive amount of iMSC exclusively from one single iPSC master clone, eliminating the major concerns in primary MSC applications, such as donor-based variability, limited sources, compromised proliferation and prone to senescence (Ozay et al., 2019; Saetersmoen et al., 2019).

This very unique potential enables the generation of millions of “off-the-shelf” copies under Good Manufacturing Practice (GMP) procedures for further therapeutic applications to treat complex and multifactorial diseases. This outstanding property enables their preferential commercial applications beyond other types of primary MSC with their guaranteed quality control.

## Discussion

Isolated and purified primary MSC have inherent greater variability due to donor diversity, tissue sources, and different culture systems, hence obtaining stable, uniform, and functionally clear MSC subtypes is the essential prerequisite to generate MSC medication.

The studies and findings about iPSC based on cell reprogramming have significantly improved our understanding of stemness maintenance and the underlying molecular mechanisms, meanwhile, they have provided a potential infinite source of MSC. For years, we and other groups have been dedicated in the studies of differentiating iPSC into iMSC to explore their therapeutic effects in multiple diseases and complications (McGrath et al., 2019; Bloor et al., 2020; Fernandez-Rebollo et al., 2020; Luo et al., 2020). It has shown that iMSC are more stable in molecular signature, proliferation capacities, tissue repair and differentiation applications than primary MSC derived from other types of tissues (Fernandez-Rebollo et al., 2020). Although iMSC are generated from iPSC, there is no need to worry about the inherit of unstable genome or their carcinogenesis potential since now iPSC can be induced without virus plasmids or tumorigenic gene c-myc. This modification and improvement have greatly push forward the clinical iMSC applications in treating different types of diseases.

Emerging data have demonstrated that iMSC possess tremendous potential for industrialized large-scale production to obtain stable and sustainable “off-the-shelf” products to meet clinical needs (McGrath et al., 2019; Ozay et al., 2019; Saetersmoen et al., 2019; Bloor et al., 2020; Chen et al., 2020). They can be continually and endlessly differentiated and generated from infinite iPSC that harvested from individual skin fibroblasts or other cell types from peripheral blood and other tissues, eliminating ethic-related issues. However, to better compare the therapeutic efficacy between iMSC and the primary MSC, the in-depth functionality demonstration in biological research and the underlying mechanisms are urgently needed, which provides reference to optimize their phenotype and efficacy for targeting specific diseases.

Overall, the further applications of MSC treatment depends on the comprehensive understanding of the MSC heterogeneity and their functional subgroups. Ideally, by adopting the technology platform of single-cell omics analysis and other long-term clinical research programs, such as transplantation immune regulation, hematopoiesis, autoimmune inflammation, neural, cartilage and skin regeneration and repair of various animal models, it is able to describe the changes of functional subsets of iMSC in the process of their occurrence, maturation, passage expansion and aging, and by differentiating the superior functional subsets, it is available to identify the key regulatory genes including cell surface markers characterizing their heterogeneity, which could be applied to establish and optimize the technology for obtaining high-homogeneous MSC for specific clinical applications.

## Data Availability Statement

The original contributions presented in the study are included in the article/supplementary material, further inquiries can be directed to the corresponding author/s.

## Author Contributions

JZ: conception and design, manuscript writing, and the figure-making. MC: conception and design, and the manuscript writing. JL, CC, YL, AP, and YZ: reference selection and the manuscript drafting. GZ: conception and design, the manuscript writing, financial support, and final approval of the manuscript. All authors contributed to the article and approved the submitted version.

## Conflict of Interest

JL and AP were employed by the company Senotherapeutics Ltd. CC and YL were employed by the company Cheerland Danlun Biopharma Co., Ltd. YZ was employed by the company Lungene Biotech Ltd. The remaining authors declare that the research was conducted in the absence of any commercial or financial relationships that could be construed as a potential conflict of interest.

## Publisher’s Note

All claims expressed in this article are solely those of the authors and do not necessarily represent those of their affiliated organizations, or those of the publisher, the editors and the reviewers. Any product that may be evaluated in this article, or claim that may be made by its manufacturer, is not guaranteed or endorsed by the publisher.

## Footnotes

1
www.clinicaltrials.gov
